# Differences of Starch Granule Distribution in Grains from Different Spikelet Positions in Winter Wheat

**DOI:** 10.1371/journal.pone.0114342

**Published:** 2014-12-16

**Authors:** Anling Yu, Yong Li, Yingli Ni, Weibing Yang, Dongqing Yang, Zhengyong Cui, Zhenlin Wang, Yanping Yin

**Affiliations:** 1 National Key Laboratory of Crop Biology, Ministry of Science and Technology, Agronomy College of Shandong Agricultural University, Tai’an, Shandong, P. R. China; 2 Agricultural Products Quality Inspection Center of Jining, Jining, Shandong, P. R. China; 3 Agricultural Bureau of Rencheng District, Jining City of Shandong Province, Jining, Shandong, P. R. China; Agroecological Institute, China

## Abstract

Wheat starch development is a complex process and is markedly difference by changes in spikelet spatial position. The present study deals with endosperm starch granule distribution and spatial position during filling development. The study was conducted with pure starch isolated from wheat (*Triticum aestivum* L.), Jimai20 and Shannong1391, at 7–35 days after anthesis (DAA). The results showed that grain number, spikelet weight and grain weight per spikelet in different spatial position showed parabolic changes. Upper spikelets had highest starch and amylose content followed by basal spikelets, then middle spikelets. The paper also suggested the volume percents of B-type and A-type granule in grain of middle spikelets were remarkably higher and lower than those of basal and upper spikelets, respectively. However, no significant difference occurred in the number percents of the two type granule. The ratio of amylase to amylopectin was positively correlated with the volume proportion of 22.8–42.8 µm, but was negatively related to the volume proportion of <9.9 µm. The results indicated that the formation and distribution of starch granules were affected significantly by spikelet position, and grains at upper and basal spikelet had the potential of increasing grain weight through increasing the volume of B-type granules.

## Introduction

Wheat (*Triticum aestivum* L.) is one of the most important food crops [1]. The starch is an important part of wheat endosperm, and it accounts for 65–75% of the final dry weight of the grain and serves as a multifunctional ingredient for the food industry [2]. The relative proportion and structure of starch constituents, amylose and amylopectin, greatly influence its end-use. Structurally, Amylose is an almost linear α-1, 4 glucan molecule that comprises 25–30% of wheat grain starch. Amylopectin is a much larger glucan polymer that is highly branched and comprises 70–75% of wheat grain starch [6], amylose to amylopectin normally occur in a ratio of ≈1∶3, by weight [7].

Starch concentration increased approximately 10% from 12 to 15 days after anthesis (DAA) in all genotypes, as well as maximum starch accumulation, occurred from 7 to 14 DAA [3]. Starch, however, usually presents as granules in the endosperm of wheat grain. Briefly, granule size distribution in wheat starch is an important factor that affecting the end-use quality [8]. It is widely acknowledged that the starch is deposited in two types of granules, B-type granules (diameter <9.9 µm) and A-type granules (diameter >9.9 µm) in mature wheat grains [9], [10], [11]. Starch deposition in endosperm occurs until maturation [12]. The A-type granules start to form in the amyloplast at about 4–5 DAA and continue to form until the end of the endosperm cell division phase [4]. The diameter of these early synthesized granules stops increasing at 19 DAA but the volume continues to increase [5]. On the other hand, the formation of B-starch granules begins about 12–14 DAA and continues to enlarge until 21 DAA [13]. A-granules contain 30–36% amylose while B-granules contain 24–27% amylase [14], [15]. Also, different size starch granules have different physical, chemical and functional properties [16], [17]. These differences result in the two starch granule types being used differently in industrial food and non-food applications. For instance, starch has several roles in the bread-making process, and starch granule size affects a range of properties [18], [19]. Using 98 hard red winter wheat cultivars and 99 hard red spring wheat cultivars as materials, Park *et al*. [8] reported that, parameters of B-granules showed many significant correlations with wheat and flour properties.

Furthermore, a wheat panicle is composed of a number of spikelets. Grain weight within a wheat spike is unevenly distributed depending upon the spatial position of kernel [20], [21]. Based on their flowering date and locations within a spike, the spikelets can be classified as superior or inferior [22], [23]. Superior grains usually exhibit a faster rate of increase in dry weight than inferior grains in wheat [24]. Low grain weight and slow grain filling of inferior grains have been attributed to a limitation in carbohydrate supply [25]. There are many explanations to the poor filling of inferior spikelets, including carbon limitation [26], [27], [28], sink capacity limitation [29], unbalance in hormonal levels [30], [31], low activities and/or gene expression of enzymes involved in sucrose-to-starch conversion [32], [33], [34], and assimilate transportation impediment [35], [36]. Recent studies have shown that low physiological activities if sink (grain) at the initial grain filling and low conversion efficiency from sucrose to starch during the active grain filling period contribute to the poor filling of inferior spikelets [37]. However, knowledge about how starch content and granule size distribution in different spikelets position is limited.

In this investigation, starch extracted from two winter wheat cultivars in different spikelets position was studied in order to realize the starch content and granule size distribution in different spikelets position of wheat, and the relationship between them. This information should contribute to the understanding of the mechanism for regulating the spatial pattern of individual grain growth within a wheat spike.

## Materials and Methods

### 1.1. Experimental design

**Figure 1:**
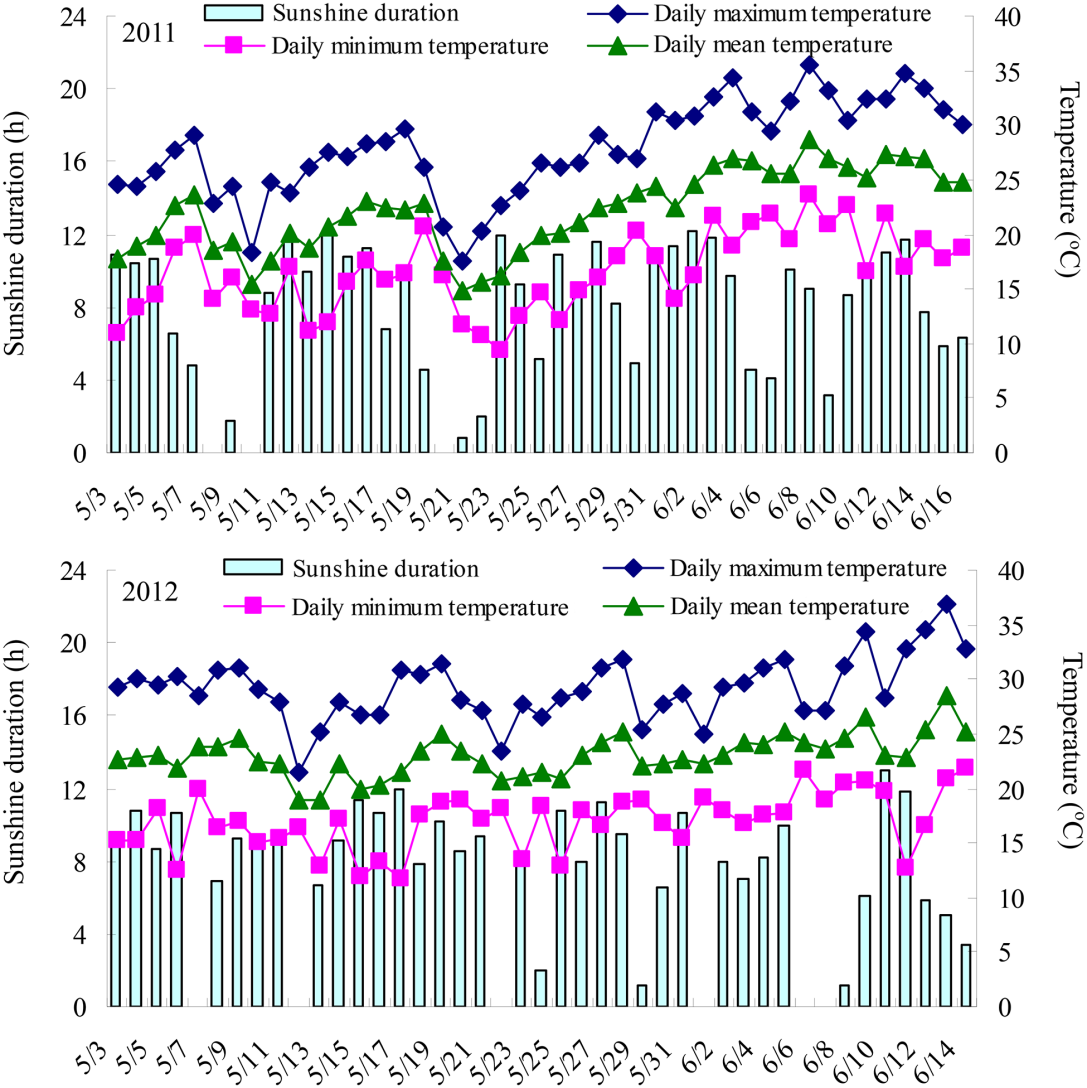
Mean daily temperature and sunshine durations from anthesis to maturity of wheat.

The field experiments were carried out in two growing seasons from 10th October 2010 to June 2011 and from 10th October 2011 to June 2012 at Tai’an Experimental Station of Shandong Agricultural University, Tai’an, China (36°09′ N, 117°09′ E). Two winter wheat (*Triticum aestivum* L.) cultivars currently used in local wheat production, Jimai20 and Shannong1391, were chosen in this study. The soil was a sandy loam and maize (*Zea mays* L.) was the previous crop. The organic matter concentration was 13.5 g kg^−1^, the total nitrogen was 0.89 g kg^−1^, and the available nitrogen, phosphorus and potassium concentrations in the soil were 77.8, 26.6 and 75.2 mg kg^−1^ in the 0–20 cm soil layer, respectively. In addition, basal fertilizer was applied at the rate of 120 kg N ha^−1^, 175 kg P_2_O_5 _ha^−1^, 150 kg K_2_O ha^−1^ before planting. Another 120 kg N ha^−1^ was top-dressed at the jointing stage (GS31) [38]. The experiments were arranged in a randomized complete design with four replications for each cultivar. The plot size was 3 m×3 m with 10 rows (0.25 m between rows) with the seeds density of 180 plants m^−2^, and the yield was harvested on 16th June 2011 and 14th June 2012. Pests, diseases, and weeds were controlled by appropriate chemical applications during crop cycle. Other cultural practices followed were according to the precise high-yielding cultivation system of Yu [39]. The mean temperatures and sunshine durations from anthesis to maturity in the two growing seasons were shown in Fig. 1.

### 1.2. Sampling

At anthesis, 100 spikes in each regimen flowering on the same date of the double central rows were labeled with red thread in order to maintain uniformity. At maturity, wheat kernels were harvested for starch granules size analysis. The spike was divided into three parts as follow: the basal spikelets (basal the first to fifth spikelets), middle spikelets (from sixth to sixteenth spikelets), the upper spikelets (seventeenth to top spikelets), respectively. Only the first and second grains in each spikelet were used. In addition, the first and second basal grains on each spikelet were detached to assay in this study.

### 1.3. Starch isolation and purification

Starch was extracted from the wheat cultivars according to the methods of Malouf and Hoseney [40] and Peng et al. [14], [15], with some modifications as follows: wheat kernels (2 g) were covered with 30 ml double distilled water at 4°C for 24 h. The softened seeds were degermed and ground with a pestle and mortar in double-distilled water until essentially all starch granules had been released. The slurry was filtered through a 74 µm screen and centrifuged at 1700 *g* for 10 min to sediment the crude starch. The crude starch was purified three times using 5 ml of 2 M NaCl, 2 mg NaOH g^−1^, 20 mg SDS g^−1^ and double-distilled water. To remove water, the starch was washed once with acetone, air-dried at room temperature (18°C) and stored at −20°C.

### 1.4. Particle size analysis

The particle size characteristics of the starch were determined using a LS13320 laser diffraction particle size analyser (Beckman Coulter, USA). About 50 mg of starch was weighed into 10 ml (Eppendorf) tubes and suspended with 5 ml of double-distilled water. The tubes were vortexed and kept at 4°C for 1 h, during which time the tubes were vortexed every 15 min. Then 1 ml of the starch suspension was transferred into the dispersion tank of a laser diffraction particle size analyser containing double-distilled water for size measurements. The assessments of equivalent volume, equivalent surface area and number proportions of starch granule were made automatically by the laser diffraction particle size analyser and represented the diameter of a sphere with the same volume as the starch grain and the diameter of a circle with the same projected area as the real starch grain. Amylose and amylopectin contents in wheat grain were determined with a coupled spectrophotometer assay. 100 mg of milled grain was stirred with 10 ml of 0.5 M KOH for 15 min at 100°C and then diluted to a volume of 50 ml with distilled water. Of this solution, 2.5 ml were diluted with 30 ml distilled water and adjusted to pH 3.5 with 0.1 mol HCL L^−1^, and then 0.5 ml of I_2_-KI reagent was added to the solution, which was diluted with distilled water to a final volume of 50 ml. After blending for 20 min, the mixture was monitored with a UV-2450 Shimadzu spectrophotometer at 471, 553, 632 and 740.3 nm. Standard amylose and amylopectin were purified from wheat. The absorption peaks of purified amylase which reacted with the I_2_-KI reagent were 632 and 471 nm, whereas those of amylopectin were 740.3 and 553 nm.

### 1.5. Data analysis

The statistical model included sources of variation due to 2 yr, Duncan’s new multiple range test was employed after a preliminary F-test to assess differences between the treatment means at the 0.05 probability level. All determinations were replicated three times, the experimental and data were performed using SPSS statistical procedures.

## Results

### 2.1. Differences in Experimental Factors

Analysis of variance for the kernel number per spikelet (KN), weight per spikelet (WS), grain weight per spikelet (GW), starch content (ST), amylase content (AM), volume distribution of starch granules <9.9 (V1), volume distribution of starch granules 9.9–22.8 (V2), volume distribution of starch granules 22.8–42.8 (V3), number distribution of starch granules <0.6 (N1), number distribution of starch granules 0.6–2.8 (N2), number distribution of starch granules 2.8–9.9 (N3) made it possible to identify the sources of variation (Table 1). Year (Yr) main effects and C×Yr, P×Yr interaction failed to influence those traits suggesting Year (Yr) was not a significant factor in the total reaction (Table 1). Cultivar (C) and Position (P) main effects were significantly affected those traits suggesting they were important to determine the quality and yield of grain starch. It also suggested the interaction was a complicated network (Table 1).

**Table 1:** Mean square significance among treatments and interactions for test factors.

variation	KN	WS	GW	ST	AM	V1	V2	V3	N1	N2	N3
Cultivar (C)	ns	**	ns	**	*	**	ns	*	*	*	ns
Position (P)	**	**	**	**	**	**	ns	**	*	*	ns
Year (Yr)	ns	ns	ns	ns	ns	ns	ns	ns	ns	ns	ns
C×P	ns	**	**	ns	ns	ns	ns	ns	*	**	ns
C×Yr	ns	ns	ns	ns	ns	ns	ns	ns	ns	ns	ns
P×Yr	ns	ns	ns	ns	ns	ns	ns	ns	ns	ns	ns

*Significantly different at the 0.05 probability level.

**Significantly different at the 0.01 probability level.

† Parameters were: kernel number per spikelet (KN), weight per spikelet (WS), grain weight per spikelet (GW), starch content (ST), amylase content (AM), volume distribution of starch granules <9.9 (V1), volume distribution of starch granules 9.9–22.8 (V2), volume distribution of starch granules 22.8–42.8 (V3), number distribution of starch granules <0.6 (N1), number distribution of starch granules 0.6–2.8 (N2), number distribution of starch granules 2.8–9.9 (N3).

‡ Not significant at *P* = 0.05 (ns).

§ Position–Year interaction (P×Yr), Cultivar–Year interaction (C×Yr), Cultivar-Position interaction (C×P).

### 2.2. Spatial distribution of grain number, spikelet weight and grain weight

**Figure 2:**
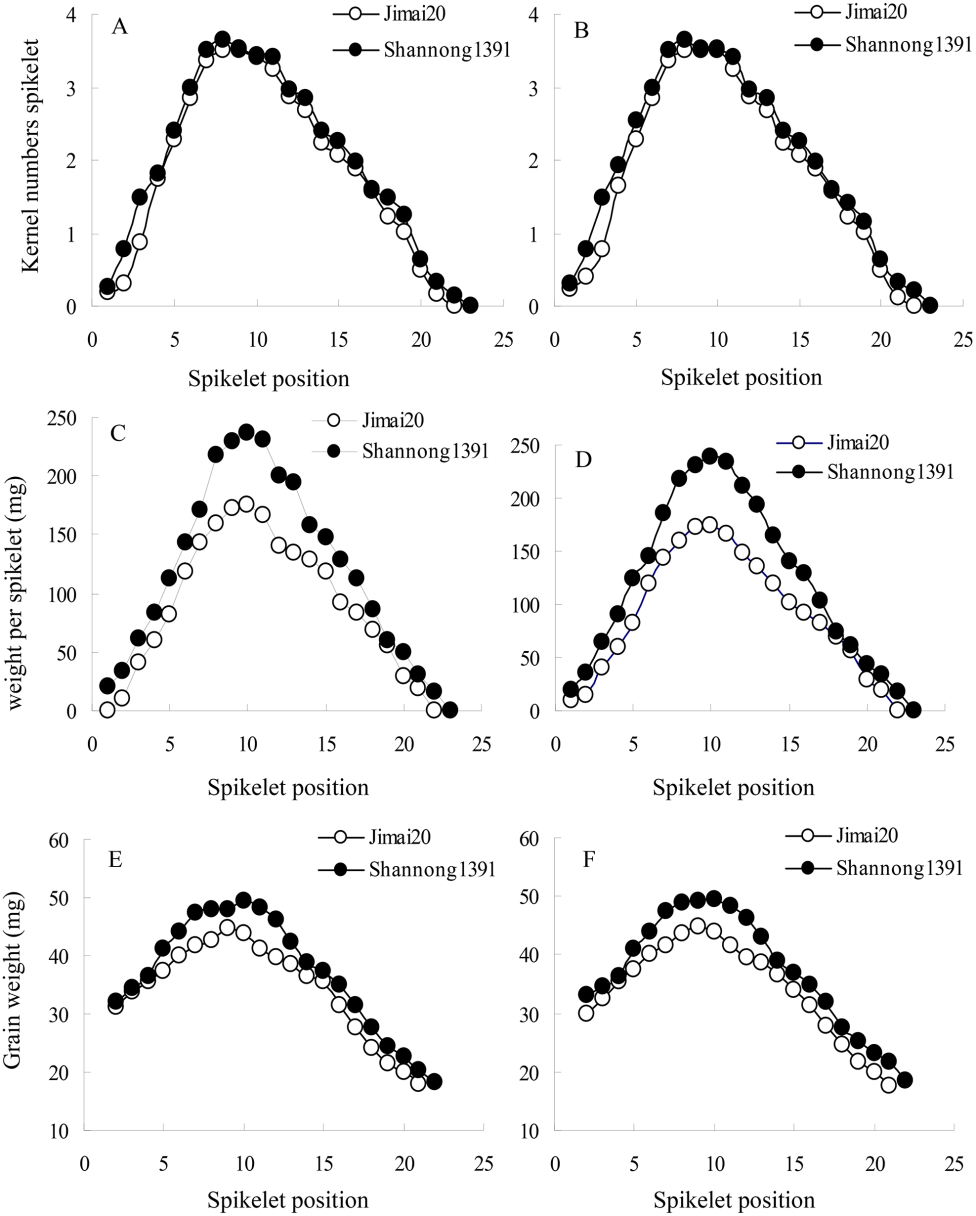
Kernel numbers per spikelet (A and B), weight per spikelet (C and D), and grain weight per spikelet (E and F) in winter wheat in growing seasons 2010/2011 (left) and 2011/2012 (right).

The grain number (Fig. 2A, B), spikelet weight (Fig. 2C, D) and grain weight (Fig. 2E, F) with the spikelet positions from the bottom to the top increased first and then decreased, showed parabolic change. And this was according with medial dominant in grain development. The spikelet weight and grain weight in different positions were showed the trend: upper spikelet < basal spikelet < middle spikelet.

### 2.3. Starch content and accumulation

**Figure 3:**
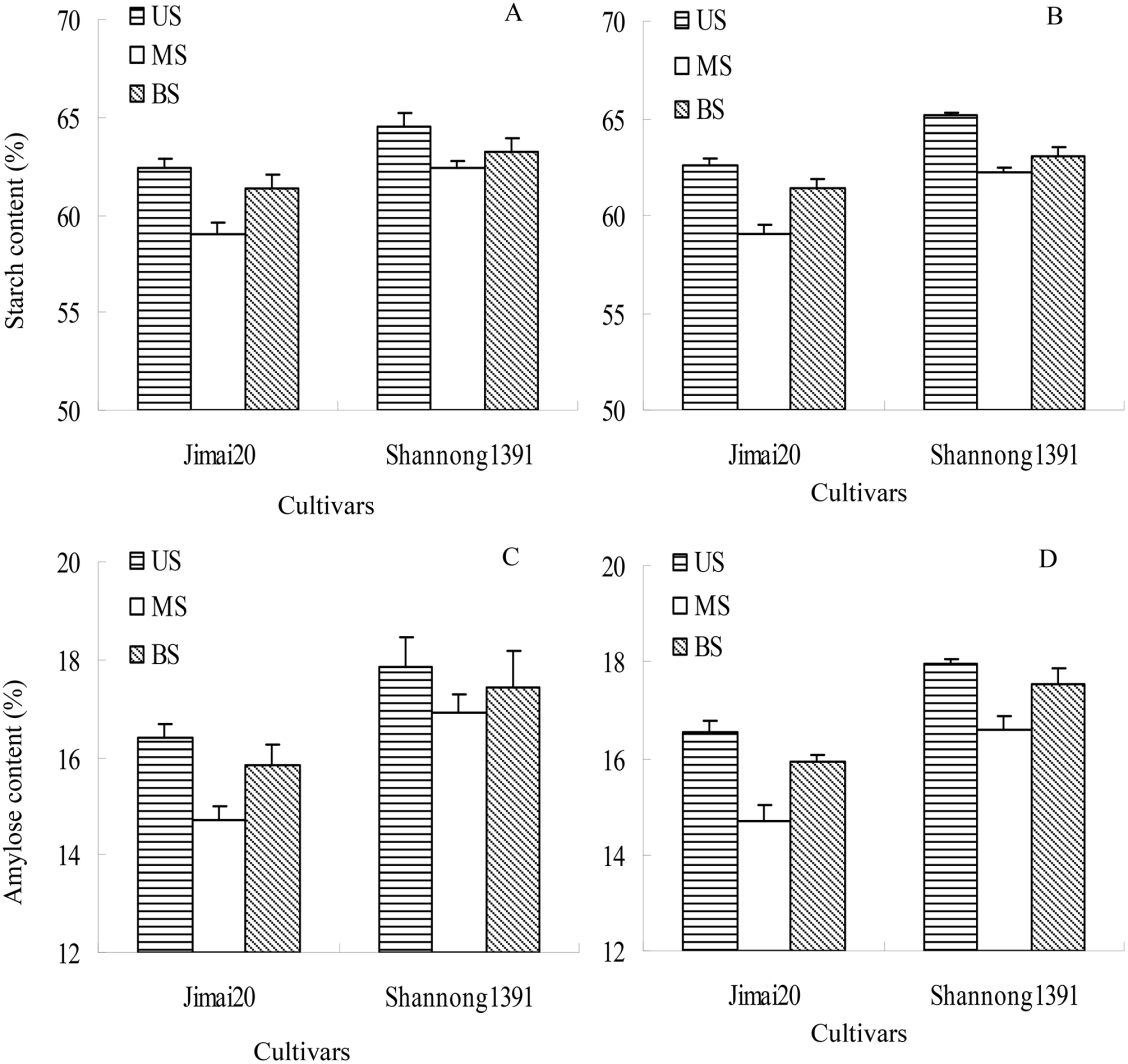
Effect of Spikelet position on starch content (A and B) and amylose content (C and D) in wheat grains in winter wheat in growing seasons 2010/2011 (left) and 2011/2012 (right). US, upper spikelets; MS, middle spikelets; BS, basal spikelets.

**Figure 4:**
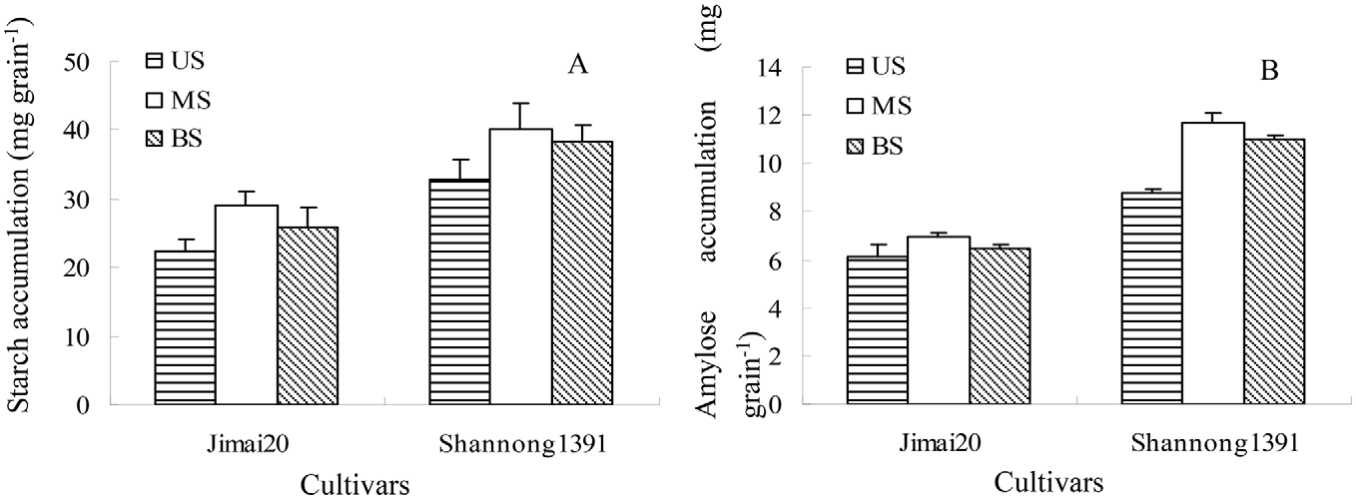
Effect of spikelet position on starch content (A) and amylase (B) accumulation in wheat grain in the growing season 2010/2011. US: Upper spikelets; MS: Middle spikelets; BS: Basal spikelets.

There were significant differences in starch and amylose content in grains differing in spikelet (Fig. 3). Upper spikelets had the highest starch and amylose content followed by basal spikelets, whereas middle spikelets had the lowest. Starch and amylose accumulation showed the reverse form with their contents (Fig. 4).

### 2.4. Starch granule distribution

#### 2.4.1. Volume distribution

The volume distribution of starch granules is given in Table 2. For the two cultivars, upper spikelets and middle spikelets had the highest and the lowest percentage of starch granules 22.8–42.8 µm, respectively. However, for Jimai20, the volume percentage of starch granules of 9.9–22.8 µm showed upper spikelets > middle spikelets > basal spikelets; but there was no significant difference between the three in Shannong1391. For the two cultivars, middle spikelets had the highest the volume percentage of starch granules of <2.8 µm and 2.8–9.9 µm, followed by basal spikelets, whereas upper spikelets had the lowest. The results showed that middle spikelets had the highest percentage of B-type starch granules.

**Table 2:** Effect of Spikelet position on the proportion by volume distribution of starch granules in wheat grain (d.f. = 17).

Seasons	Cultivars	Spikelet positions	Particle equivalent diameter of starch granule (µm)				
			<2.8	2.8–9.9	<9.9	9.9–22.8	22.8–42.8
2010/11	Jimai20	US	0.107bc	0.308c	0.415c	0.287b	0.308b
		MS	0.128a	0.342a	0.472a	0.289b	0.239a
		BS	0.112b	0.324b	0.436b	0.320a	0.244c
	Shannong1391	US	0.093bc	0.252c	0.345c	0.308a	0.347a
		MS	0.112a	0.280a	0.392a	0.304a	0.304c
		BS	0.101b	0.273ab	0.374b	0.305a	0.321b
2011/12	Jimai20	US	0.110b	0.308c	0.418c	0.285c	0.297a
		MS	0.124a	0.339a	0.463a	0.306b	0.231c
		BS	0.113b	0.321b	0.434b	0.317a	0.249b
	Shannong1391	US	0.108a	0.234c	0.342c	0.307a	0.351a
		MS	0.088b	0.302a	0.390a	0.306a	0.304c
		BS	0.102ab	0.275b	0.377b	0.308a	0.315b
Comparison of all grain positions			P<0.05	P<0.01	P<0.01	P<0.05	P<0.05
s.e.d.			0.0022	0.0024	0.0035	0.0026	0.0024

Means within cultivar followed by a different letter are significantly different at *P*<0.05. US, Upper spikelets; MS, Middle spikelets; BS, Basal spikelets.

#### 2.4.2. Number distribution

Proportions of starch granules <2.8 µm and <9.9 µm were in the ranges of 96.4%–96.6% and 99.9% of the total number, respectively (Table 3), which showed that number of granules in wheat grain analyzed was made up of B-type starch granules. These results implied that small starch granules are the main component although they contribute less to the total volume. Spikelets exerted little effect on the alteration of percentage of starch granules >2.8 µm. However, it was showed basal spikelets > middle spikelets > upper spikelets for the percentage of starch granules <0.6 µm. On the contrary, for the percentage of starch granules 0.6–2.8 µm, middle spikelets were the highest, followed by upper spikelets, and the basal spikelets were lowest, which showed that it is more conducive to the development of small starch granule in the middle spikelets.

**Table 3:** Effect of Spikelet position on the proportion by number distribution of starch granules in wheat grain (d.f. = 17).

Seasons	Cultivars	Spikelet positions	Particle equivalent diameter of starch granule (µm)				
			<0.6	0.6–2.8	<2.8	2.8–9.9	<9.9
2010/11	Jimai20	US	0.416b	0.549b	0.965a	0.034a	0.99a
		MS	0.412c	0.553a	0.965a	0.034a	0.99a
		BS	0.421a	0.545c	0.966a	0.033a	0.99a
	Shannong1391	US	0.405b	0.560ab	0.965a	0.034a	0.99a
		MS	0.401c	0.563a	0.964a	0.035a	0.99a
		BS	0.415a	0.550b	0.965a	0.034a	0.99a
2011/12	Jimai20	US	0.418b	0.547b	0.965a	0.034a	0.99a
		MS	0.411c	0.554a	0.965a	0.034a	0.99a
		BS	0.425a	0.541c	0.966a	0.033a	0.99a
	Shannong1391	US	0.407b	0.558b	0.965a	0.034a	0.99a
		MS	0.401c	0.563a	0.964a	0.035a	0.99a
		BS	0.419a	0.546c	0.965a	0.034a	0.99a
Comparison of all grain positions			P<0.01	P<0.01	NS	NS	NS
s.e.d.			0.018	0.016	0.002	0.01	0.001

Means within cultivar followed by different letter are significantly different at *P*<0.05. US: Upper spikelets; MS: Middle spikelets; BS: Basal spikelets.

### 2.5. Starch content

Starch content and starch component content at maturity were significant different among the grains in different spikelet positions (Table 4). For both cultivars, the 3 parameters as starch, amylase and amylopectin in the different grains had a trend of upper spikelet > basal spikelet > middle spikelet. However, amylose/amylopectin (AM/AP) in grains had different trends between the two cultivars. In Jimai20, AM/AP in upper spikelet was the highest, followed by that in basal spikelet, and the middle spikelet was the lowest. In addition, for Shannong1391, the middle spikelet was the lowest, but there were no significant differences between upper spikelet and basal spikelet.

**Table 4:** Effect of Spikelet position on the proportion of starch and its components in wheat grain.

Seasons	Cultivars	Spikelet positions	ST (/100)	AM(/100)	AP(/100)	AM/AP
2010/11	Jimai20	US	62.41a	16.42a	45.99a	0.357a
		MS	59.01c	14.71c	44.30b	0.332c
		BS	61.34b	15.82b	45.52a	0.347b
	Shannong1391	US	64.55a	17.84a	46.71a	0.382a
		MS	62.39c	16.91c	45.48b	0.372b
		BS	63.18b	17.45ab	45.73b	0.381a
2011/12	Jimai20	US	62.57a	16.55a	46.02a	0.359a
		MS	59.11c	14.71c	44.40b	0.331c
		BS	61.41b	15.91b	45.50a	0.349b
	Shannong1391	US	65.15a	17.95a	47.71a	0.380a
		MS	62.15c	16.61c	45.54b	0.364b
		BS	63.12b	17.54ab	45.58b	0.383a
Comparison of all grain positions			P<0.01	P<0.05	P<0.05	P<0.05
s.e.d.			0.71	0.45	0.81	0.029

Means within cultivar followed by different letter are significantly different at *P*<0.05. US: Upper spikelets; MS: Middle spikelets; BS: Basal spikelets; ST, starch; AM, amylase; AP, amylopectin; AM/AP, amylose/amylopectin.

**Figure 5:**
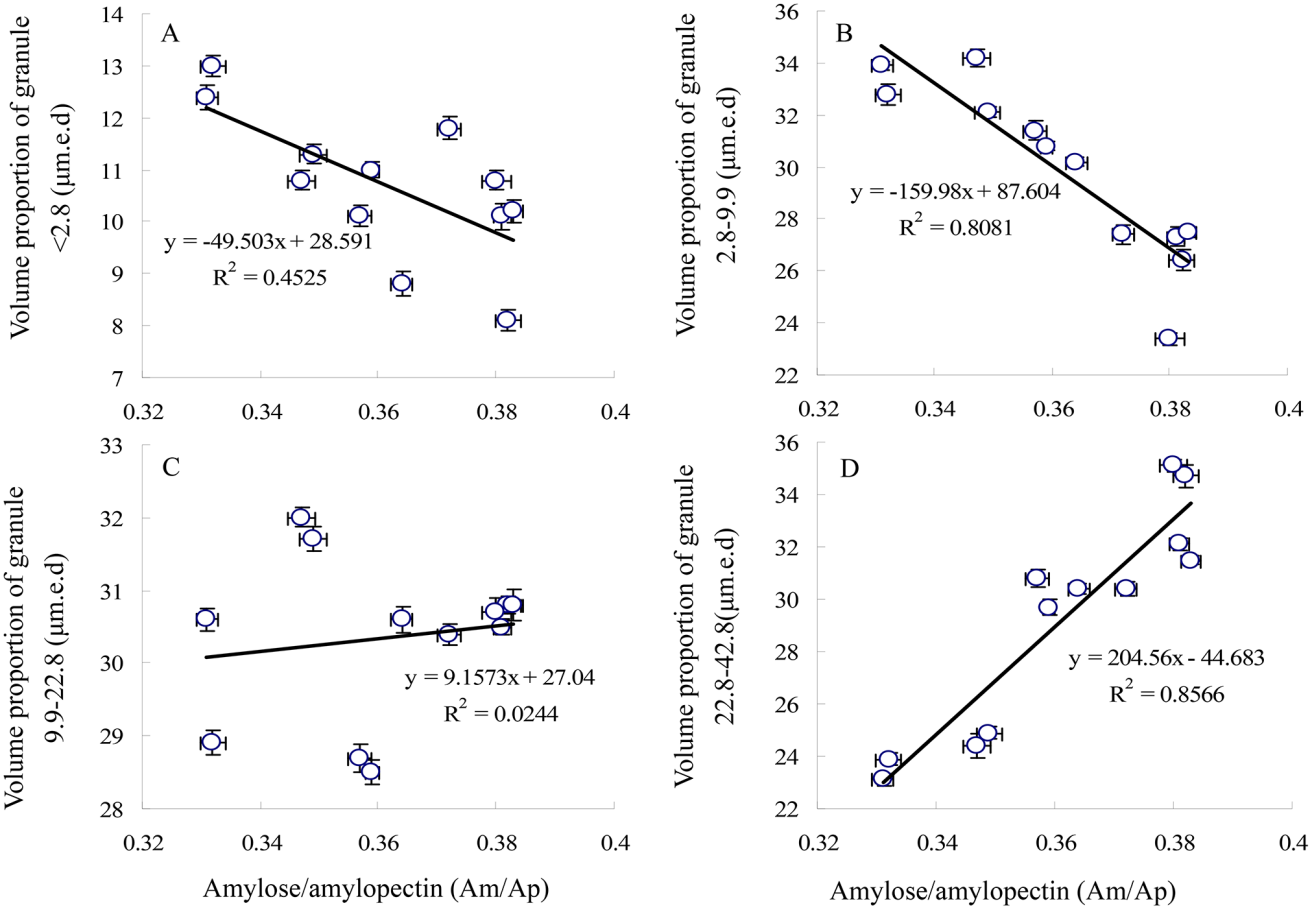
The relationship between distribution by volume of starch and the ratio of amylose to amylopectin granules in grain.

The proportion of granules by volume (volume proportion) of starch granules <2.8 µm and 2.8–9.9 µm were negatively correlated with the ratio of amylose to amylopectin (r = −0.67, P<0.05; r = −0.89, P<0.01; respectively; Fig. 5A and B). A significantly positive correlation (r = 0.92, P<0.01; Fig. 5D) was found between volume proportions of starch granules 22.8–2.8 µm and the ratio of amylose to amylopectin. Volume proportions of starch granules 9.9–22.8 µm were not significantly correlated with the ratio of amylose to amylopectin (r = −0.15; P>0.05; Fig. 5C).

Grain weight at different spikelet position was significantly different (Table 1). And, a positively correlated was founded with Grain weight at different spikelet position and the volume proportion of starch granules <9.9 µm. A negatively correlation was found between grain weight and the volume proportion of starch granules 22.8–42.8 µm, while no significant relationships were observed with the volume proportion of starch granules 9.9–22.8 µm. However, there was no significant relationship between grain number and the number proportion of starch granules (Table 5).

**Table 5:** Correlation coefficients between grain weight and the proportion of volume distribution and number distribution of starch granules at different spikelet positions.

Cultivars	Volume diameter of starch granule (µm)			Number diameter of starch granule (µm)		
	<9.9	9.9–22.8	22.8–42.8	<0.6	0.6–2.8	<2.8
Jimai20	0.90**	0.56	−0.98**	−0.20	0.25	0.14
Shannong1391	0.90**	0.64	−0.98**	−0.12	0.22	0.19

## Discussion

It has been well reported in the literature that the degree and rate of grain filling in wheat spikelets differ largely with their positions on a panicle [30]. Markedly differences were also reported in the floret development, carbohydrate supply and endogenous hormones and starch synthesis among grains within a rice and wheat spike [16], [41]. Nevertheless, results of previous studies mainly took the sucrose content as a limiting factor for starch synthesis in inferior grains, further reason for this observation was unclear and needed to be investigated [42], [43]. For instance, Jiang *et al*. [25] suggested that superior grains had higher sucrose contents (substrate of starch synthesis) at early and middle grain filling compared to inferior ones. However, Zhang *et al*. [44] found that the source supply was abundant during grain filling stage, while the sink size and ability of assimilation conversion were the main limiting factors for starch accumulation. Based on the present study and previous work, we proposed that the starch accumulation was much higher in middle spikelets (the 6^th^ to 16^th^ spikelet) which behaved preferential growth and stronger positions compared with basal and upper spikelets. It was also confirmed the results of Tan *et al*. [45].

Environment conditions affected starch granules distribution obviously. The number of B-type starch granules decreased when exposed to higher temperatures, correspondingly, that of A-type starch granules increased [46], [47]. Similar observation had been reported in heat stress and shade studies [48]. In a comparison of irrigated conditions, rain-fed conditions resulted in the increase of the volume and surface area percents of B-type starch granules, correspondingly, those of A-type starch granules were reduced. Li *et al*. [49] reported that shading led to a significant reduction in the proportions of B-type starch granules volume distribution, while increased in those of A-type starch granules. It behaved the opposite of P fertilization [50]. We now proposed that the middle spikelets had significant higher percentage of B-type starch granules compared with basal and upper spikelets. Firstly, the B-type granules formed within protrusions from A-type amyloplasts [51]. Secondly, sufficient substrate was propitious to small starch granules polarization and development [52]. Finally, the limited substrate for starch accumulation was mainly partitioned for growing starch granules, not for producing more starch granule numbers in grain of basal and upper spikelets.

Wheat A- and B-type starch granules are reported to have significantly different chemical compositions such as amylose, amylopectin, lipid, and protein concentrations [7], [12], [53]. Thus, starch granule size distribution had a close association with its component content. Various studies reported amylose content was higher in the large starch granules than the small granules. It was suggested that the A-type granules have a 4–10% higher amylose content than the B-type granules [15], [54], while Ames *et al*. [55] found only minor differences (2–3%) in amylose content. Raeker *et al*. [56] reported that the starch content was negatively correlated with volume percentage of granule <5 and 9.9 µm. Upper spikelets had the highest amylose content followed by basal spikelets, whereas middle spikelets had the lowest. Integrating with starch granule size distribution, we suggested that amylose content in grain was positively correlated to volume percentage of A-type granule (*R* = 0.96, *P*<0.01).

This study also showed that number percentage of starch granules 2.8–9.9 µm (midsize) and >9.9 µm (large) in grains had no significant difference among upper, middle and basal spikelets (Table 1). However, the variability in number distribution of the small starch granules (<2.8 µm) among different spikelet position was highly significant. Number percentage of granules 0.6–2.8 µm in grains of middle spikelets was remarkably higher than that of basal and upper spikelets, indicating that it was more conducive to the development of small starch granules in middle spikelets. It was resulted in high substrate supply and degradation capacity and high activities of enzymes involving in starch synthesis, especially the development of B-type granules in middle spikelets.

## Conclusion

In this study, the development of starch granules in grain with spikelet position was investigated. Grain weight was positively correlated with the volume proportion of <9.9 µm (*R* = 0.90**), but remarkable negative correlations were found between grain weight and the volume proportion of 22.8–42.8 µm. (*R* = −0.98**). There were significantly positive relationship between AM/AP and the volume proportion of 22.8–42.8 µm and negative correlation between AM/AP and the volume proportion of <9.9 µm. Thus, the middle spikelets had significant higher percentage of B-type starch granules, yet, the limited substrate for starch accumulation was mainly partitioned for growing starch granules of basal and upper spikelets.

## References

[1] Rome (2009) Food and Agriculture Organization of the United Nations (FAO). 6086142

[2] Abdel-Aal ESM, Hucl P, Chibbar RN, Han HL, Demeke T (2002) Physicochemical and structural characteristics of flours and starches from waxy and non-waxy wheats. Cereal Chem 79:458–464.

[3] Laudencia-Chingcuanco DL, Stamova BS, You FM, Lazo GR, Beckles DM, Anderson OD (2007) Transcriptional profiling of wheat caryopsis development using cDNA microarrays. Plant Mol Biol 63:651–668. 17211515 10.1007/s11103-006-9114-y

[4] Dai Z (2010) Activities of enzymes involved in starch synthesis in wheat grains differing in starch content. Russ J Plant Physiol 57:74–78.

[5] Geetika A, Sarita J, Pierre H, Ravindra NC (2014) Wheat genome specific granule-bound starch synthase I differentially influence grain starch synthesis. Carbohydr Polym 114:87–94. 25263868 10.1016/j.carbpol.2014.08.004

[6] Hurkman WJ, McCue KF, Altenbach SB, Korn A, et al (2003) Effect of temperature on expression of genes encoding enzymes for starch biosynthesis in developing wheat endosperm. Plant Sci 164:873–881.

[7] Soh HN, Sissons MJ, Turner MA (2006) Effect of starch granule size distribution and elevated amylose content on durum dough rheology and spaghetti cooking quality. Cereal Chem 83:513–519.

[8] Park SH, Wilson JD, Seabourn BW (2009) Starch granules size distribution of hard red winter and hard red spring wheat, its effects on mixing and bread making quality. J Cereal Sci 49:98–105.

[9] Soulaka AB, Morrison WR (1985) The amylose and lipid content, dimensions, and gelatinization characteristics of some wheat starches and their A- and B-granule fractions. J Sci Food Agr 36:709–718.

[10] Vermeylen R, Goderis B, Reynaers H, Delcour JA (2005) Gelatinisation related structural aspects of small and large wheat starch granules. Carbohydr Polym 62:170–181.

[11] Kim HS, Huber KC (2008) Channels within soft wheat starch A- and B-type granules. J Cereal Sci 48:159–172.

[12] Dengate HN (1984) Swelling, pasting, and gelling of wheat starch. In: Pomeranz, Y. (Ed.), Adv cereal sci technol. AACC, USA 49–82.

[13] Bechtel DB, Zayas I, Kaleikau L, Pomeranz Y (1990) Size distribution of wheat starch granules during endosperm development. Cereal Chem 67:59–63.

[14] Peng M, Hucl P, Chibbar RN (2001) Isolation, characterization and expression analysis of starch synthase I from wheat (Triticum aestivum L.). Plant Sci 161:1055–1062. 11081966

[15] Peng M, Gao M, Abdel-Aal ESM, Huel P, et al (1999) Separation and characterization of A- and B-type starch granules in wheat endosperm. Cereal Chem 76:375–379.

[16] Chiotelli E, Meste ML (2002) Effect of small and the large wheat starch granules on thermo mechanical behavior of starch. Cereal Chem 79:286–293.

[17] Park SH, Wilson JD, Chung OK, Seib PA (2004) Size distribution and properties of wheat starch granules in relation to crumb grain score of pup-loaf bread. Cereal Chem 81:699–704.

[18] Sahlstrom S, Baebre AB, Brathen E (2003) Impact of starch properties on hearth bread characteristics. II. Purified A- and B-granule fractions. J Cereal Sci 37:285–293.

[19] Sahlstrom S, Brathen E, Lea P, Autio K (1998) Influence of starch granule size distribution on bread characteristics. J Cereal Sci 28:157–164.

[20] Calderini DF, Reynolds MP (2000) Changes in grain weight as a consequence of de-graining treatments at pre- and post- anthesis in synthetic hexaploid lines of wheat. Aus J Plant Physiol 27:183–191.

[21] Pan J, Jiang D, Cao WX, Sun CF (2005) Effects of spikelet and grain positions on grain number, weight and protein content of wheat spike. Acta Agron Sin 31:431–437.

[22] Stamp PH, Geisler G (1976) Grain development in relation to grain position in two spring wheat cultivars. Z. Acker- und Pflanzenbau 142:264–274.

[23] Stoddard FL (1999) Variation in grain mass, grain nitrogen, and starch B-granule content within wheat heads. Cereal Chem 76:139–144.

[24] Liang TB, Yin YP, Cai RG, Yan SH, et al (2008) Differences of starch accumulation and related enzyme activities between superior and inferior grains of wheat cultivars with large spike. Acta Agron Sin 34:150–156.

[25] Jiang D, Cao WX, Dai TB, Jing Q (2003) Activities of key enzymes for starch synthesis in relation to growth of superior and inferior grains on winter wheat spike. Plant growth regul 41:247–257.

[26] Murty PSS, Murty KS (1982) Spikelet sterility in relation to nitrogen and carbohydrate contents in rice. Ind J Plant Physiol 25:40–48.

[27] Sikder HP, Gupta DKD (1976) Physiology of grain in rice. Ind Agric 20:133–141.

[28] Wang Y (1981) Effectiveness of supplied nitrogen at the primordial panicle stage on rice characteristics and yields. Int Rice Res Newsl 6:23–24.

[29] Kato T (2004) Effect of spikelet removal on the grain filling of Akenohoshi, a rice cultivar with numerous spikelets in a panicle. J Agric Sci 142:177–181.

[30] Yang J, Zhang J (2006) Grain filling of cereals under soil drying. N phytol 169:223–236. 10.1111/j.1469-8137.2005.01597.x 16411926

[31] Zhang H, Tan G, Yang L, Yang J, et al (2009) Hormones in the grains and roots in relation to post-anthesis development of inferior and superior spikelets in japonica/indica hybrid rice. Plant Physiol Biochem 47:195–204. 19117763 10.1016/j.plaphy.2008.11.012

[32] Ishimaru T, Hirose T, Matsuda T, Goto A, et al (2005) Expression patterns of genes encoding carbohydrate-metabolizing enzymes and their relationship to grain filling in rice (Oryza sativa L.): Comparison of caryopses located at different positions in a panicle. Plant Cell Physiol 46:620–628. 15701658 10.1093/pcp/pci066

[33] Jeng TL, Wang CS, Chen CL, Sung JM (2003) Effects of grain position on the panicle on starch biosynthetic enzyme activity in developing grains of rice cultivar Tainung67 and its NaN_3_-induced mutant. J Agric Sci 141:303–311.

[34] Wang E, Wang J, Zhu X, Hao W, et al (2008) Control of rice grain-filling and yield by a gene with a potential signature of domestication. Nat Genet 40:1370–1374. 18820698 10.1038/ng.220

[35] Yang J, Zhang W, Wang Z, Liu L, Zhu Q (2002) Source-sink characteristics and the translocation of assimilates in new plant type and inter sub specific hybrid rice. Agric Sci Chin 1:155–162.

[36] Yang J, Zhang J (2010) Crop management techniques to enhance harvest index in rice. J Exp Bot 61:3177–3189. 20421195 10.1093/jxb/erq112

[37] Yan S, Li W, Yin Y, Wang Z (2010) Sink strength in relation to growth of superior and inferior grains within a wheat spike. J Agric Sci 148:567–578.

[38] Zadoks J C, Chang TT, Konzak CF (1974) A decimal code for the growth stages of cereals. Weed Res 14:415–421.

[39] Yu SL (1990) Wheat in Shandong Province. Chin Agric Press, BJ, Chin.

[40] Malouf RB, Hoseney RC (1992) Wheat hardness. I. A method to measure endosperm tensile strength using tablets made from wheat flour. Cereal Chem 69:164–168.

[41] Yang JC, Su BL, Wang ZQ, Lang YZ, Zhu QS (1998) Characteristics and physiology of grain-filling in inter-subspecific hybrid rice. Sci Agric Sinica 31:7–14.

[42] Wang Z, Yin Y, He M, Zhang Y, et al (2003) Allocation of photosynthates and grain growth of two wheat cultivars with different potential grain growth in response to pre- and post-anthesis shading. J Agron Crop Sci 189:280–285.

[43] Wang F, Cheng F, Zhang G (2006) The relationship between grain filling and hormone content as affected by genotype and source-sink relations. Plant Growth Regul 49:1–8.

[44] Zhang QY, Liu N, Jin J, Liu XB, et al (2000) Relationship between starch, protein accumulation and substrate supply during grain filling in spring wheat. J of Tritic Crops 20:55–58.

[45] Tan XS, Bi JJ, Wang JH, Ye BX (2012) Differences of starch granules in grains from different spikelet position and their correlation with grain weight in winter wheat. Acta Agron Sin 11:1809–1816.

[46] Macleod LC, Duffus CM (1988) Temperature effects on starch granules in developing barley grains. J Cereal Sci 8:29–37.

[47] Yan SH, Yin YP, Li WY, Dai ZM, et al (2008) Effect of high temperature during grain filling on starch accumulation, starch granule distribution, and activities of related enzymes in wheat grains. Acta Agron Sin 34:1092–1096.

[48] Dai ZM, Yin YP, Zhang M, Li WY, et al (2008) Starch granule size distribution in wheat grains under irrigated and rainfed conditions. Acta Agron Sin 34:795–802.

[49] Li W, Yan S, Yin Y, Wang Z (2010) Starch granule size distribution in wheat grain in relation to shading after anthesis. J Agric Sci 148:183–189.

[50] Ni Y, Wang Z, Yin Y, Li W, et al (2012) Starch granule size distribution in wheat grain in relation to phosphorus fertilization. J Agric Sci 150:45–52.

[51] Parker ML (1985) The relationship between A-type and B-type starch granules in the developing endosperm of wheat. J Cereal Sci 3:271–278.

[52] Li WY, Yan SH, Yin YP, Li Y, Liang T, et al (2009) Starch granule size distribution and starch component content in wheat grain in relation to shading stress after anthesis. Acta Ecolog Sin 29:298–306.

[53] Seib PA (1994) Wheat starch: Isolation, structure and properties. Oyo Toshitsu Kagak 41:49–69.

[54] Shinde SV, Nelson JE, Huber KC (2003) Soft wheat starch pasting behavior in relation to A- and B-type granule content and composition. Cereal Chem 80:91–98.

[55] Ames NP, Clarke JM, Maningat O, Izydorczyk MS (1999) Variation of starch granule size in durum wheat cultivars. AACC International: St. Paul, MN.

[56] Raeker MO, Gaines CS, Finney P L, Donelson T (1998) Granule size distribution and chemical composition of starches from 12 soft wheat cultivars. Cereal Chem 75:721–728.

